# Slight acceleration in podocyte mRNA loss in preterm-born children aged 3–5 years

**DOI:** 10.1007/s00467-025-06983-z

**Published:** 2025-10-08

**Authors:** Zhengqi Cui, Chao Ning, Junling Ma, Lulu Zhang, Xueou Liu, Li Kong, Ying Chang, Fangrui Ding

**Affiliations:** 1 Department of Neonatology, Tianjin Central Hospital of Obstetrics and Gynecology, Tianjin, China; 2 Xiangya School of Medicine, Central South University, Hunan, China; 3 Tianjin Key Laboratory of Human Development and Reproductive Regulation, No. 156 Nan Kai San Ma Lu, Tianjin, China; 4 Department of Neonatology, Nankai University Maternity Hospital, Tianjin, China; 5 Research Institute of Obstetrics and Gynecology, Tianjin Central Hospital of Obstetrics and Gynecology, Tianjin, China; 6 Prenatal Diagnosis Center, Tianjin Central Hospital of Obstetrics and Gynecology, Tianjin, China

**Keywords:** Preterm birth, Podocyte, Chronic kidney disease (CKD)

## Abstract

**Background:**

The global survival rate of preterm infants has been progressively increasing. However, concerns regarding their long-term prognosis persist. This study aimed to investigate podocyte mRNA loss in 3–5-year-old full-term and preterm children to elucidate the role of podocyte depletion in the pathogenesis of chronic kidney disease (CKD) in preterm infants.

**Methods:**

A total of 80 children aged 3–5 years, born at Tianjin Central Hospital of Gynecology and Obstetrics, were included in this study: 42 preterm infants (gestational age 24–29 weeks) and 38 full-term infants. Morning urine samples were collected to examine podocyte mRNA levels (expressed as the urinary podocin mRNA-to-creatinine ratio, UpodCR), urine protein, and urine albumin levels. The impact of perinatal factors on UpodCR was also analyzed.

**Results:**

Results indicated that the rate of podocyte mRNA loss in the preterm group was significantly higher than in the full-term group (1.54-fold). No significant differences were observed in urine protein and urine albumin levels between the two groups. Perinatal factor analysis revealed that gestational age and antenatal corticosteroid use were significant risk factors for podocyte loss in childhood.

**Conclusions:**

This study is the first to confirm accelerated podocyte loss in the urine of 3–5-year-old preterm children. Although less severe than in the early postnatal period, it remains higher than in full-term children, providing crucial evidence for the involvement of podocyte depletion in the pathogenesis of preterm-related CKD. It also underscores the need for careful evaluation of the benefits and risks associated with antenatal corticosteroid use.

**Graphical abstract:**

**Figure:**
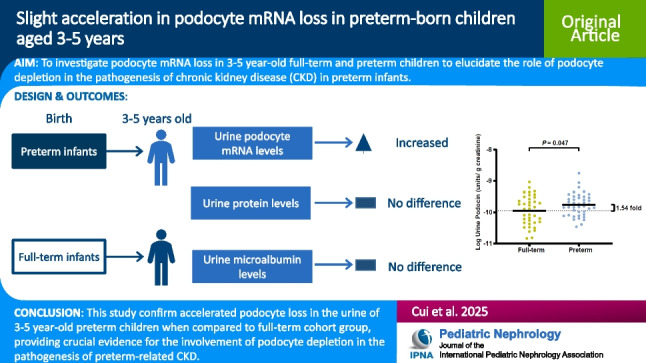
A higher resolution version of the Graphical abstract is available as Supplementary information

**Supplementary Information:**

The online version contains supplementary material available at 10.1007/s00467-025-06983-z.

## Introduction

The survival rate of preterm infants has gradually improved worldwide, but we need to not only focus on early survival issues but also consider long-term prognosis [1, 2]. In recent years, clinical studies have increasingly confirmed that preterm birth, especially extreme preterm birth, is a significant risk factor for the occurrence of various systemic diseases in the long term [3–7]. The incidence of neurological disorders, respiratory system diseases, metabolic disorders and so on significantly increases among extremely preterm infants [3–7]. In the field of kidney disease, research in recent years has similarly shown that preterm infants have a significantly higher incidence of chronic kidney disease (CKD) in childhood and adulthood when compared to full-term infants [3, 8, 9].

There is an expanding body of research dedicated to elucidating the mechanisms underlying the development of long-term CKD resulting from preterm birth [10–20]. The prevailing hypothesis, known as the “nephron number hypothesis”, posits that preterm infants with reduced nephron numbers are at a higher risk for kidney diseases later in life [10–14]. Kidney immaturity is evident in preterm infants at birth, and preterm delivery can disrupt normal development, leading to a diminished nephron endowment. Consequently, this reduction in nephron quantity increases susceptibility to kidney disease [10–14]. Furthermore, recent studies from our laboratory suggest that “podocyte depletion hypothesis” also plays a significant role in this process. Our previous research indicates that preterm birth accelerates podocyte differentiation, resulting in a decrease in the overall count of differentiated podocytes [15–20]. In our animal studies, we conducted long-term follow-up and observed persistent acceleration of podocyte loss in preterm animals [19]. The continuous loss of podocytes, along with the enlargement of both podocytes and glomeruli and a decrease in podocyte density within the kidney, suggests a gradual increase in the risk for the development and progression of CKD [19].

In our previous human study, we observed comparable findings. Specifically, preterm infants with corrected gestational age exceeding 37 weeks (equivalent to full-term gestational age) exhibited a significantly higher loss of podocytes compared to full-term infants, reaching approximately 3–5 times greater levels [17–19]. Nephrogenesis in most individuals is completed around 36 weeks of gestation in humans, and the timing for selection in our previous study on preterm infants was based on this milestone. At this specific time point, there was a markedly higher urinary podocyte loss compared to full-term infants [17–19]. However, these results suggest only a potential increased risk for future development of CKD, rather than providing strong evidence. Follow-up data in preterm infants were insufficient to determine whether there was persistent podocyte loss during their growth. Additionally, there are distinct differences between humans and animal models, such as maternal factors, dissimilarities in neonatal resuscitation techniques, oxygen management, mechanical ventilation procedures, and other medication administrations. Therefore, we conducted long-term follow-up studies on preterm infants, collecting urine samples to test for podocyte loss, urinary protein levels, and albuminuria levels, in order to explore the role of podocyte depletion in the mechanism of CKD development among preterm infants.

## Methods

The present study was approved by the Institutional Review Board of Tianjin Central Hospital of Gynecology and Obstetrics (Approval Number: 2020KY049, 2024KY066). All research activities adhered strictly to the guidelines outlined in the Declaration of Helsinki. Prior to their children's participation in the study, written informed consent was obtained from all parents or legal guardians.

### Participants

The present study included children 3–5 years old who were born at Tianjin Central Hospital of Gynecology and Obstetrics. As for children born preterm, infants born between 2016 and 2018 at gestational age 24–29 weeks were recruited. After excluding children with known kidney diseases, chromosomal abnormalities, congenital anomalies, acute infections at the time of collection, a history of asphyxia or acute kidney injury, or neonatal sepsis, full-term children aged 3 to 5 years were age-matched and included as controls in the present study. Gestational age at birth was identified from the medical records based on maternal report of last menstrual period or ultrasound estimation. Other general information was obtained from the medical records.

### Urine sample collection and processing

This method has been utilized in previous studies [21, 22] and a standardized protocol was uniformly applied throughout all urine processing procedures. Urine samples were collected from September 2021 to November 2021. Urine samples were collected to examine podocyte mRNA levels, urine protein, and urine albumin levels. Morning urine specimens were collected in 50 mL sterile containers, ensuring a minimum volume of 30 mL. Upon collection, the samples were immediately subjected to centrifugation at 4 °C for 15 min at a speed of 3220 relative centrifugal force. Two milliliters of the resulting supernatant were preserved at –20 °C for later analysis of urinary protein and creatinine concentrations. The levels of urinary protein and albumin were quantified by an automated biochemical analyzer (Mindray BS-480) using the prepared supernatant. The sediment obtained from the urine samples was reconstituted twice in 750 µL of chilled diethylpyrocarbonate-treated phosphate-buffered saline (DEPC-PBS), which had been cooled to 4 °C beforehand, and then transferred to a 2.0 mL Eppendorf tube. This suspension was subsequently centrifuged at 13,523 rpm for 5 min at 4 °C. After this centrifugation step, the pellet was resuspended in RLT buffer supplemented with β-mercaptoethanol (at a ratio of 10 μL per mL of RLT buffer) following the procedure detailed in Qiagen’s RNeasy Mini Kit (catalog number 74106). The resuspended pellet was promptly subjected to RNA isolation following the protocol outlined in the RNeasy Mini Kit (catalog number 74106) provided by Qiagen. RNA extraction was carried out as per the guidelines included with the Qiagen RNeasy Mini Kit (catalog number 74106). Following this, the absolute quantification of podocin mRNA levels was determined using the TaqMan Fast Universal PCR Master Mix (catalog number 4352042; Applied Biosystems) in a reaction volume of 20 μL for each cDNA sample. The experiment employed a TaqMan probe specific to human *NPHS2* (podocin) (catalog number Hs00922492_m1; Applied Biosystems) on the Applied Biosystems 7500 Fast Real-Time PCR System. For accurate quantification, standard curves were constructed for each assay through serial dilutions of cDNA standards with known concentrations of human podocin cDNA sequences, enabling molar-based data analysis for each probe. Ultimately, podocin mRNA levels were adjusted relative to creatinine concentration and reported as the urinary podocin mRNA-to-creatinine ratio (UpodCR).

### Statistical analysis

Continuous variables were tested for normality using the Shapiro–Wilk test. Data was reported as mean ± SD for normally distributed variables and median (range) for non-normally distributed variables. Categorical variables were summarized as frequencies and percentages. Group comparisons for normally distributed continuous variables were performed using the independent-sample t-test, whereas the Wilcoxon Rank Sum test was used for non-normally distributed continuous variables. Categorical variables were compared using the Chi-square test. A *P*-value < 0.05 was considered statistically significant. All statistical analyses were conducted using R software (version 4.2.2).

## Results

### Basic clinical information of children born full-term and preterm

In this study, a total of 80 children aged 3–5 years were enrolled, including 38 children born full-term and 42 age-matched children born preterm. Baseline clinical information is summarized in Table 1. As shown in Table 1, no significant differences were observed between the two groups in terms of gender, mode of delivery, height at the time of urine collection, maternal gestational diabetes, or gestational hypertension. However, significant differences were noted in preterm-related parameters such as gestational age, birth weight, birth height, 1-min Apgar score, antenatal corticosteroid use, administration of ibuprofen, and neonatal morbidity. Additionally, there were significant differences in weight at the time of urine collection, which may be attributed to the poorer nutritional status of preterm infants. Furthermore, maternal age was higher in the preterm group compared to the full-term group.

**Table 1:** Characteristics of full-term and preterm born children aged 3 to 5 years

Analyzed children	Full-term (≥ 37 weeks)	Preterm (< 30 weeks)	*P* value
Number of children, *n* (%)	38 (47.5)	42 (52.5)	NA
Gestational age, weeks, median (range)	39.4 (37.7–40.4)	27.1 (24.1–29.3)	< 0.001 *
Male, *n* (%)	17 (44.7)	23 (54.8)	0.502
Vaginal delivery, *n* (%)	21 (55.3)	27 (64.3)	0.552
Birth weight, kg, mean ± SD	3.4 ± 0.3	1.0 ± 0.2	< 0.001 *
Birth height, cm, median (range)	50.0 (48.0–51.0)	36.0 (32.5–39.0)	< 0.001 *
1 min Apgar score, median (range)	10 (10–10)	6 (1–9)	< 0.001 *
Age at time point of urine collection, years, median (range)	4 (3–5)	4 (3–5)	0.649
Weight at time point of urine collection, kg, median (range)	17.8 (15.0–23.4)	17.0 (13.0–22.0)	0.015 *
Height at time point of urine collection, cm, median (range)	106.0 (96.0–125.0)	105.0 (90.0–115.0)	0.140
Maternal age, years, median (range)	32 (28–36)	33 (27–41)	0.045 *
Antenatal corticosteroids use, *n* (%)	1 (2.6)	27 (64.3)	< 0.001 *
Maternal gestational hypertension, *n* (%)	0 (0.0)	2 (4.8)	0.519
Maternal gestational diabetes, *n* (%)	0 (0.0)	2 (4.8)	0.519
BPD, *n* (%)	0 (0.0)	19 (45.2)	< 0.001 *
Neonatal intracranial hemorrhage, *n* (%)	0 (0.0)	26 (61.9)	< 0.001 *
ROP, *n* (%)	0 (0.0)	22 (52.4)	< 0.001 *
Neonatal infection, *n* (%)	0 (0.0)	19 (45.2)	< 0.001 *
Administration of ibuprofen, *n* (%)	0 (0.0)	18 (42.9)	< 0.001 *

*n*, number; %, percent; *SD*, standard deviation; *BPD*, bronchopulmonary dysplasia; *ROP*, retinopathy of prematurity; *NA*, not available

Asterisks (*) indicate statistically significant differences between the two groups (*P* < 0.05)

### Podocyte mRNA loss in children born preterm

**Fig. 1:**
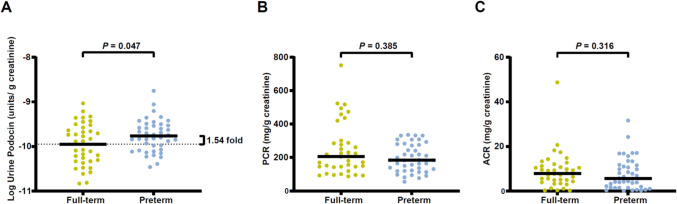
The urine podocyte loss level in preterm infants was 1.54 times that of full-term infants at 3–5 years of age. Urine podocin mRNA levels, protein-to-creatinine ratio (PCR) and albumin-to-creatinine ratio (ACR) were compared between preterm (*n* = 42) and full-term (*n* = 38) infants. (**A**) Preterm infants exhibited a 1.54-fold (95% confidence limits 1.01–2.36) increased podocin mRNA in the urine pellet compared to full-term infants (preterm vs. full-term: 1.734E-10 units vs. 1.125E-10 units, *P* = 0.047). Solid black horizontal lines indicated the mean values for each group. Student’s *t*-test was used for analysis. (**B**, **C**) No significant differences were observed in PCR (*P* = 0.385) or ACR (*P* = 0.316) between preterm and full-term infants. Solid black horizontal lines indicated the median values for each group. The Wilcoxon Rank Sum test was used for comparison

Urinary podocin mRNA was examined to assess podocyte loss in all children. As shown in Fig. 1A, the preterm group exhibited a significantly higher rate of podocyte loss compared to the full-term group, with a magnitude 1.54-fold greater than that of the full-term group.

### Urine protein and urine albumin in children born preterm

In addition to urine podocyte detection, urine protein and urine albumin levels were also detected. After being normalized by urine creatinine, protein-to-creatinine ratio (PCR) and albumin-to-creatinine ratio (ACR) were compared between preterm and full-term groups. As shown in Fig. 1B–C, there is no difference between children born preterm and full-term.

### Perinatal factors related to podocyte mRNA loss

Preterm infants are frequently exposed to a range of perinatal factors. In addition to gestational age, we examined the impact of various perinatal factors on UpodCR, an indicator of podocyte loss. Univariate regression analysis results for each perinatal factor in relation to podocyte loss are presented in Table 2. Furthermore, multivariate analysis was conducted, revealing that gestational age and antenatal corticosteroid use were significant risk factors for podocyte loss during childhood (Table 3).

**Table 2:** Univariate linear regression analysis of perinatal risk factors and urinary podocyte loss

Variable	Range	*β* (95% CI)	*P* value
Gestation (weeks)	24.1–40.4	− 0.014 (− 0.029; 0.001)	0.072
Sex	1 = male; 2 = female	0.148 (− 0.036; 0.332)	0.119
Delivery mode	1 = vaginal delivery; 2 = cesarean delivery	− 0.041 (− 0.231; 0.149)	0.674
Birth weight (kg)	0.7–4.0	− 0.077 (− 0.155; 0.000)	0.053
Birth height (cm)	32.5–51.0	− 0.011 (− 0.024; 0.002)	0.097
1 min Apgar score	1–10	− 0.020 (− 0.056; 0.016)	0.283
Age at urine collection (years)	3–5	− 0.115 (− 0.254; 0.024)	0.109
Weight at urine collection (kg)	13.0–23.4	− 0.024 (− 0.066; 0.018)	0.261
Height at urine collection (cm)	90.0–125.0	− 0.012 (− 0.025; 0.002)	0.088
Maternal age (years)	27–41	0.001 (− 0.030; 0.031)	0.972
Antenatal corticosteroids use	1 = yes; 2 = no	− 0.125 (− 0.319; 0.069)	0.211
Maternal hypertension	1 = yes; 2 = no	− 0.196 (− 0.792; 0.401)	0.522
Mothers with diabetes, *n* (%)	1 = yes; 2 = no	0.051 (− 0.547; 0.649)	0.868
BPD	1 = yes; 2 = no	− 0.060 (− 0.279; 0.159)	0.593
Neonatal intracranial hemorrhage	1 = yes; 2 = no	− 0.050 (− 0.249; 0.149)	0.625
ROP	1 = yes; 2 = no	0.067 (− 0.142; 0.276)	0.531
Neonatal infection	1 = yes; 2 = no	− 0.089 (− 0.307; 0.130)	0.428
Administration of ibuprofen	1 = yes; 2 = no	− 0.162 (− 0.382; 0.059)	0.155

*BPD*, bronchopulmonary dysplasia; *ROP*, retinopathy of prematurity

**Table 3:** Multivariate linear regression analysis of perinatal risk factors and urine podocyte loss

Variable	Range	*β* (95% CI)	*P* value
Gestation (weeks)	24.1–40.4	− 0.130 (− 0.250; − 0.010)	0.037 *
Sex	1 = male; 2 = female	0.207 (− 0.007; 0.421)	0.063
Delivery mode	1 = vaginal delivery; 2 = cesarean delivery	− 0.152 (− 0.369; 0.065)	0.175
Birth weight (kg)	0.7–4.0	− 0.370 (− 0.905; 0.165)	0.180
Birth height (cm)	32.5–51.0	0.133 (− 0.004; 0.270)	0.062
1 min Apgar score	1–10	0.065 (− 0.026; 0.156)	0.166
Age at urine collection (years)	3–5	− 0.124 (− 0.388; 0.140)	0.359
Weight at urine collection (kg)	13.0–23.4	− 0.043 (− 0.123; 0.037)	0.293
Height at urine collection (cm)	90.0–125.0	0.012 (− 0.019; 0.043)	0.450
Maternal age (years)	27–41	− 0.033 (− 0.073; 0.007)	0.108
Antenatal corticosteroids use	1 = yes; 2 = no	− 0.427 (− 0.825; − 0.029)	0.040 *
Maternal hypertension	1 = yes; 2 = no	0.301 (− 0.532; 1.134)	0.481
Mothers with diabetes, *n* (%)	1 = yes; 2 = no	− 0.055 (− 0.752; 0.642)	0.878
BPD	1 = yes; 2 = no	− 0.022 (− 0.470; 0.426)	0.922
Neonatal intracranial hemorrhage	1 = yes; 2 = no	0.378 (− 0.196; 0.952)	0.201
ROP	1 = yes; 2 = no	0.350 (− 0.071; 0.771)	0.108
Neonatal infection	1 = yes; 2 = no	− 0.344 (− 0.875; 0.187)	0.209
Administration of ibuprofen	1 = yes; 2 = no	0.128 (− 0.254; 0.510)	0.513

*BPD*, bronchopulmonary dysplasia; *ROP*, retinopathy of prematurity

Asterisks (*) denote perinatal factors significantly associated with urine podocyte loss (*P* < 0.05)

## Discussion

To the best of our knowledge, this study is the first to evaluate podocyte loss in individuals born preterm during childhood. Previous clinical studies have indicated that preterm birth is a significant risk factor for the development of CKD both in childhood and adulthood [3, 8, 9]. The nephron hypothesis is widely regarded as the primary mechanism involved in this process [10–14]. Moreover, our previous studies demonstrated that podocyte loss was significantly higher in preterm infants during the early postnatal period compared to full-term infants [17–19]. However, it remains unclear whether this accelerated podocyte loss continues into later childhood or adulthood, which would help determine if the increased risk of preterm-related CKD in later stages is driven by podocyte depletion. To further elucidate the role of podocyte depletion in the pathogenesis of preterm-related CKD, we collected urine samples from preterm children aged 3 to 5 years. The primary finding of this study is that there is accelerated podocyte loss in the urine of children born preterm compared with those born full-term, providing critical evidence for the involvement of podocyte depletion in the mechanisms underlying preterm-related CKD.

The extent of podocyte loss in urine among children born preterm aged 3–5 years differs significantly from that observed in the early postnatal period when compared to the full-term group. In the early postnatal period, when preterm infants reach a corrected gestational age of over 37 weeks, podocyte loss is approximately 3 to 5 times higher than in full-term infants [17–19]. In this study, we focused on children born at a gestational age of less than 30 weeks. Our previous research has shown that lower gestational age correlates with increased podocyte loss, indicating that podocyte loss in this cohort is more severe, potentially exceeding 3–5 times higher than in full-term infants in the early postnatal period [18]. Currently, at around 3–5 years of age, podocyte loss in preterm children is approximately 1.5 times higher than in full-term children, which is less pronounced compared to the early postnatal stage. This suggests that kidney developmental processes partially normalize during the growth period of children. From the perspective of kidney development, nephrogenesis is largely completed around 36 weeks of gestation in full-term infants. In preterm infants, nephrogenesis concludes earlier than in full-term infants, resulting in fewer nephrons and differentiated podocytes compared to full-term infants [17, 23]. Therefore, two potential explanations for the recovery or reversal of podocyte loss can be proposed. First, the number of podocytes may increase over time, as supported by studies confirming podocyte regeneration [24–27]. However, definitive conclusions require kidney biopsy, which poses practical challenges in clinical settings. Another possibility is that podocytes undergo compensatory changes during early childhood (neonatal age to 3–5 years). Although the kidneys of preterm infants complete nephrogenesis around 36 gestational weeks, with a lower number of nephrons and differentiated podocytes compared to full-term infants, the glomerular filtration membrane coverage remains insufficient, leading to higher podocyte loss. In addition, glomerular development such as an increase in glomerular size may also contribute to the larger glomerular filtration membrane coverage as well as early increase in glomerular filtration rate. During the subsequent growth and adaptation process, podocytes undergo hypertrophy to compensate for their insufficient number. This compensatory increase in size partially mitigates the loss of podocyte quantity. Consequently, children born preterm exhibit a suboptimal state characterized by higher but milder loss in podocytes. When kidneys are subjected to various adverse conditions during growth, such as obesity, infection, and aging, podocyte loss accelerates, and the compensatory capacity of the remaining podocytes gradually diminishes until it becomes insufficient to maintain normal function. Consequently, proteinuria develops, leading to kidney diseases, glomerular sclerosis, and development of CKD [28]. Our animal research primarily provides indirect evidence supporting the aforementioned second possibility [19]. Follow-up observations in our animal studies consistently demonstrated similar results: initially, the difference in urinary podocyte loss was minimal, but over time, this loss became progressively more severe. Similarly, the density of podocytes in kidney specimens from animals decreased from an initial 18% difference to a final 32% difference [19].

As an indicator of podocyte loss, previous studies have utilized various methods such as detecting podocyte-specific mRNA, extracellular vesicles, and direct counting of podocytes [22, 29, 30]. In this study, we opted for the detection of podocyte-specific mRNA in urine sediment, as numerous studies have confirmed that urinary podocin mRNA levels effectively reflect podocyte loss. With regard to extracellular vesicles, the centrifugation speed employed in this study was not sufficient to pellet them. According to the report by Hara, the collection of extracellular vesicles generally requires ultracentrifugation (100,000 g, 2 h) [29]. Therefore, urine podocin mRNA was selected for assessing podocyte loss in this study.

The result of albuminuria can also indirectly confirm our above speculation. In our previous study, we observed a statistically significant increase in albuminuria compared to the full-term group [18]. However, at ages 3–5 years, no significant difference in albuminuria was detected relative to full-term group, indicating that kidney developmental processes partially normalize. Furthermore, these findings also confirm that urinary podometrics is a sensitive method for detecting kidney abnormalities.

As shown in Table 3, in addition to gestational age, the use of antenatal corticosteroids was also identified as a high-risk factor for podocyte loss. Existing studies suggest that for preterm infants under 34 weeks, the benefits of prenatal corticosteroid use generally outweigh its adverse effects [31–37]. However, to date, there has been a lack of research examining the impact of prenatal corticosteroid use on kidney function. Our study addresses this gap by demonstrating that prenatal corticosteroid exposure is associated with increased podocyte loss, thereby posing a significant risk for kidney function impairment. Obstetricians should therefore carefully evaluate the indications for prenatal corticosteroid use, weighing both potential benefits and risks. Additionally, further studies should focus on this critical issue.

There are several limitations in the present study. Firstly, this study is a cross-sectional one and does not involve a preterm birth cohort followed from birth through childhood to adulthood. A more persuasive approach would involve an excellent longitudinal preterm birth cohort that is continuously monitored until adulthood or development of kidney diseases. Secondly, since it is difficult to obtain kidney biopsy samples of preterm infants during this stage, we can only make inferences through urine indicators. However, it is difficult to completely clarify the accurate state of kidney as well as podocytes.

## Conclusion

In this study, we investigated the loss of podocytes in a cohort of children aged 3–5 years who were born preterm with a gestational age of less than 30 weeks. Our findings revealed that podocyte loss was significantly higher in this cohort compared to the full-term group, although it was less severe than in the early stages following birth and in cases of kidney disease. These results confirm that podocyte depletion is an important mechanism underlying long-term CKD associated with preterm birth.

## Supplementary Information

Below is the link to the electronic supplementary material.

Graphical abstract (PPTX 107 KB)

## Data Availability

All data generated or analyzed during this study are included in this published article.
