# Supplementary material for: Slight acceleration in podocyte mRNA loss in preterm-born children aged 3–5 years
Source: Pediatr Nephrol. 2025 Oct 8;41(2):491–8. doi: 10.1007/s00467-025-06983-z (PMC12727743; doi:10.1007/s00467-025-06983-z)
Supplement: Supplementary file 1 — Graphical abstract (PPTX 107 KB) [file 467_2025_6983_MOESM1_ESM.pptx]

## Slide 1
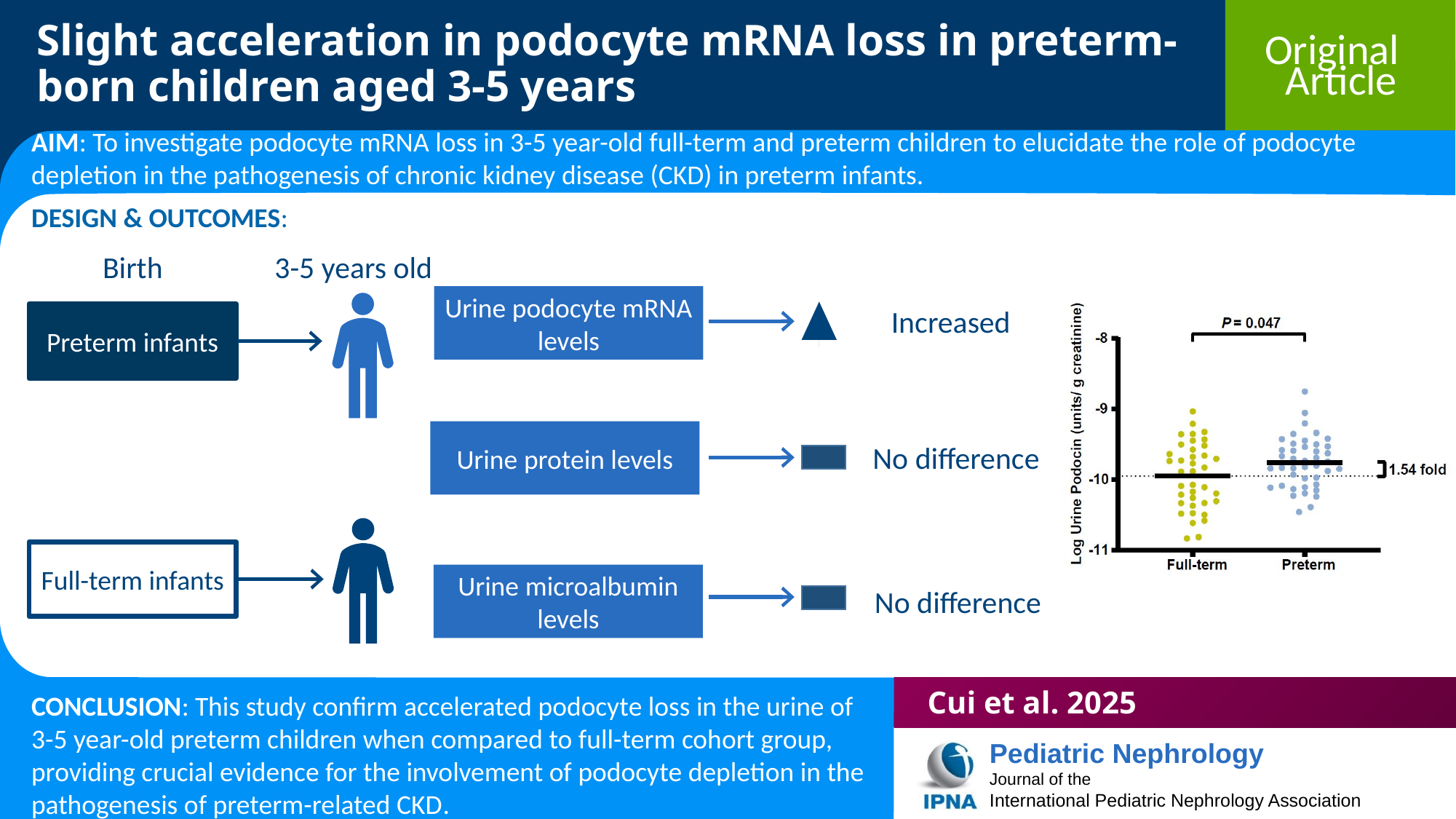

Slight acceleration in podocyte mRNA loss in preterm-born children aged 3-5 years
AIM: To investigate podocyte mRNA loss in 3-5 year-old full-term and preterm children to elucidate the role of podocyte depletion in the pathogenesis of chronic kidney disease (CKD) in preterm infants.
DESIGN & OUTCOMES:
Birth
3-5 years old
Urine podocyte mRNA levels
Increased
Preterm infants
Urine protein levels
No difference
Full-term infants
Urine microalbumin
levels
No difference
Cui et al. 2025
CONCLUSION: This study confirm accelerated podocyte loss in the urine of 3-5 year-old preterm children when compared to full-term cohort group, providing crucial evidence for the involvement of podocyte depletion in the pathogenesis of preterm-related CKD.
